# Development and Pilot Testing of PrOFILE‐ST: A Pediatric Surgical Oncology Capacity and Quality Assessment Tool for Resource‐Limited Settings

**DOI:** 10.1002/cam4.71122

**Published:** 2025-08-05

**Authors:** Fernanda Kelly Marques de Souza, Simone de Campos Vieira Abib, Rodrigo Chaves Ribeiro, Débora Rebollo de Campos, Lily Saldaña Gallo, Raul Ramirez De la Cruz, Jaime Shalkow‐Klincovstein, Jesús Ponce‐Cruz, Mohammed Elnour, Amon Ngongola, Seith Kalota, Bruce Bvulani, Logan Houston, Riya Misal, Heather Forrest, Nadya Sullivan, Alyana Alvarez, Srikanth Naradasu, Aman Maru, Miriam L. Gonzalez‐Guzman, Paola Friedrich, Abdelhafeez Abdelhafeez

**Affiliations:** ^1^ Department of Pediatric Surgery Pediatric Oncology Institute ‐ GRAACC ‐ Federal University of São Paulo São Paulo Brazil; ^2^ Department of Pediatric Surgery Barretos Children's Cancer Hospital São Paulo Brazil; ^3^ Clinical Governance & Quality Barretos Children's Cancer Hospital São Paulo Brazil; ^4^ Instituto Nacional de Salud del Niño Lima Peru; ^5^ Pediatric Surgical Oncology ABC Cancer Center Mexico City Mexico; ^6^ Pediatric Oncology ABC Cancer Center Mexico City Mexico; ^7^ National Pediatric Surgery Center Wad Madani Sudan; ^8^ Department of Pediatric Surgery University Teaching Hospital Lusaka Zambia; ^9^ Department of Pediatric Global Medicine St. Jude Children's Research Hospital Memphis Tennessee USA; ^10^ University of Rochester Medical Center, Golisano Children's Hospital Department of Surgery, Division of Pediatric Surgery Rochester New York USA

**Keywords:** capacity assessment tool, global health disparities, pediatric surgical oncology, PrOFILE‐ST

## Abstract

**Background:**

The global incidence of childhood cancer is rising, with nearly 90% of cases occurring in low‐ and middle‐income countries (LMICs), where mortality remains high due to limited access to quality pediatric surgical care. To address this, the International Society of Pediatric Surgical Oncology (IPSO) and St. Jude Children's Research Hospital developed the Pediatric Oncology Facility Integrated Local Evaluation Surgical Tool (PrOFILE‐ST) to assess surgical capacity and quality in resource‐limited settings.

**Methods:**

PrOFILE‐ST was developed in three stages: construction, content validation, and pilot testing. The tool was refined over three years through biweekly team meetings and expert feedback. Pilot testing took place at six institutions across five countries (Sudan, Zambia, Peru, Mexico, Brazil), collecting both quantitative and qualitative data on surgical practices. Data was analyzed using descriptive statistics, producing reports to guide quality improvement.

**Results:**

The final tool consists of 12 modules and 273 questions. Pilot testing revealed differences in surgical capacity between upper‐middle‐income countries (UMICs) and lower middle/low‐income countries (LMICs/LICs). UMICs had better infrastructure, while LMICs/LICs excelled in surgical reporting and timeliness. Improvement priorities included the development of pediatric surgical oncology fellowships, standardization of operative reporting, systematic morbidity and mortality analysis, and establishment of real‐time multidisciplinary team discussions.

**Conclusions:**

PrOFILE‐ST is an effective tool for assessing pediatric oncology surgery across diverse settings, highlighting strengths and areas for improvement. It can guide quality improvement efforts to address global disparities in childhood cancer outcomes.

**Abbreviations**
IPSO: International Society of Pediatric Surgical Oncology
LIC: low‐income country
LMIC: lower‐ middle income country
PrOFILE‐ST: Pediatric Oncology Facility Integrated Local Evaluation Surgical Tool
QI: quality Improvement
UMIC: upper‐middle income country
WHO: world Health Organization

## 1 Introduction

An estimated 400,000 new cases of childhood cancer are diagnosed annually, with nearly 90% of those cases occurring in low‐ and middle‐income countries (LMICs) [1, 2, 3]. Mortality in high‐income countries (HICs) is approximately 28% [4], whereas it remains substantially higher in LMICs due to challenges in delivering high‐quality pediatric oncology care [5, 6, 7, 8]. Identifying, monitoring, and treating pediatric oncology patients requiring surgical intervention is particularly difficult in resource‐limited settings [9].

Reducing disparities in pediatric cancer outcomes is a global imperative. Existing global health frameworks such as the Pediatric Oncology in Developing Countries (PODC) committee of the International Society of Pediatric Oncology (SIOP) adapted treatment guidelines, the World Health Organization (WHO) Global Initiative for Childhood Cancer (GICC), and the WHO Hospital Assessment Tool have advanced efforts to standardize pediatric oncology care delivery and assess broader institutional readiness [10, 11]. However, these instruments do not provide detailed evaluation of surgical oncology infrastructure, processes, or performance indicators.

Capacity and quality metrics have been integral to healthcare programs since the 1990s [12] demonstrating that improvements in care processes, surgical structures, and staff training enhance patient outcomes and reduce costs [13]. Benchmarking allows healthcare teams to measure internal and external performance [14, 15], yet few tools assess healthcare performance and training needs in LMICs, particularly in pediatric oncology surgery.

To address this gap, the International Society of Pediatric Surgical Oncology (IPSO) and St. Jude Children's Research Hospital collaborated to develop and validate the Pediatric Oncology Facility Integrated Local Evaluation Surgical Tool (PrOFILE‐ST). This modular, multi‐domain assessment instrument was specifically designed to evaluate pediatric surgical oncology service capacity, workforce, clinical practices, and quality metrics across diverse global settings. The present study describes the development, validation, and pilot implementation of PrOFILE‐ST and explores its potential utility as a structured platform for quality improvement in surgical care for children with cancer.

## 2 Methods

**FIGURE 1:**
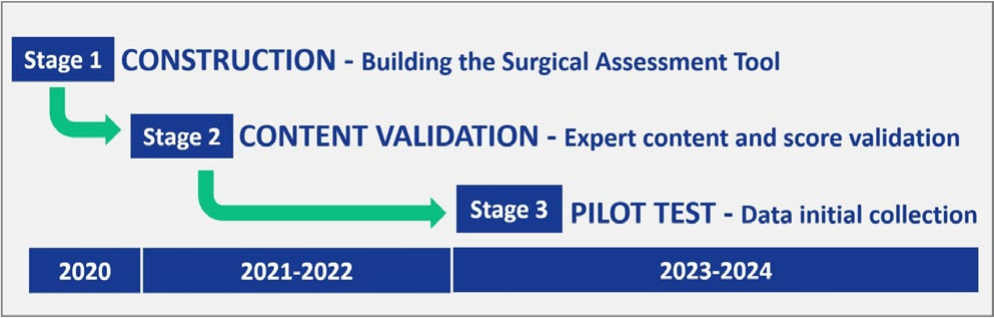
Stages of development and pilot testing PrOFILE‐ST.

This study was conducted in accordance with the ethical standards of the institutional and national research committees and with the 1964 Helsinki Declaration and its later amendments or comparable ethical standards. Ethical approval was obtained from St. Jude Children's Hospital Institutional Review Board (Protocol #00000029FWA00004775); IRB Number 21–0860. Where participants were institutions or professional staff, consent for participation and publication was also obtained. Data were anonymized prior to analysis to ensure confidentiality. PrOFILE‐ST development and pilot testing followed a structured three‐stage approach: Construction, Content Validation, and Pilot Testing (Figure 1).

### 2.1 Stage 1—Construction

PrOFILE‐ST was adapted from the original PrOFILE tool developed by the St. Jude Global Metrics and Performance Team in collaboration with IPSO [16]. The development process spanned three years, during which an expert panel conducted biweekly iterative reviews to refine the tool. The primary goal was to create a comprehensive, structured instrument to systematically evaluate pediatric surgical oncology metrics.

The final version of PrOFILE‐ST consists of 12 modules and 273 questions, incorporating multiple response formats, including multiple‐choice, Boolean, Likert scale, and numeric value questions. A scoring system ranging from 0 to 5 was integrated to facilitate quantitative comparisons across institutions. To enhance content relevance, international pediatric surgical oncology experts reviewed the tool, providing iterative feedback on its clarity, comprehensiveness, and applicability in LMIC settings.

### 2.2 Stage 2—Content Validation

Between 2021 and 2022, the tool underwent rigorous content validation through an alpha testing process involving nine internationally recognized pediatric surgical oncology experts identified through IPSO. Experts assessed the tool's clarity, comprehensiveness, usability, and scoring system through structured feedback forms and virtual discussions.

A threshold Item‐Content Validity Index (I‐CVI) of 0.78 was used to determine whether an item required revision or removal, consistent with accepted practices for expert panels of 5–10 members. Items falling below this threshold were flagged for modification. Testing was concluded when no additional items required revision and the scale‐level CVI (S‐CVI/Ave) exceeded 0.90, indicating strong overall content validity.

### 2.3 Stage 3—Pilot Testing

Pilot testing was conducted across six institutions in five countries (Sudan, Zambia, Peru, Mexico, and Brazil). Sites were selected based on their high pediatric surgical oncology patient volume and demonstrated institutional commitment to quality improvement.

At each site, the PrOFILE‐ST assessment was completed by a team led by a senior pediatric surgeon, often in collaboration with other surgical specialists, a pediatric oncologist, a nurse, and a quality officer to ensure comprehensive and accurate responses.

**FIGURE 2:**
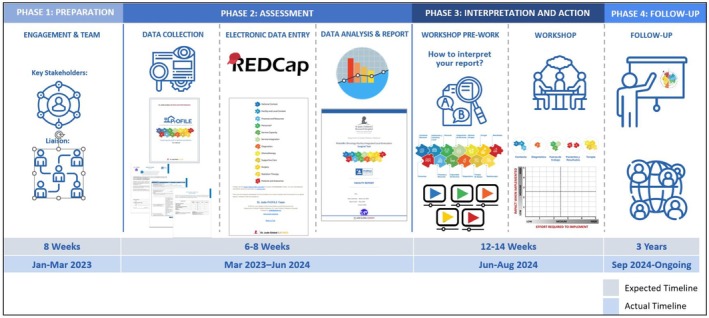
Implementation Timeline for PrOFILE‐ST pilot testing.

The pilot testing process followed a standardized three‐phase model (Figure 2):

Preparation Phase: Each institution received a detailed user guide, recorded walkthrough sessions, and live support meetings by the coordinating team. These resources ensured standardized understanding and implementation of the tool, and enhanced reproducibility across institutions in diverse settings. The survey was administered using REDCap, a secure, web‐based application designed for research data collection.

Assessment Phase: Each institution completed the PrOFILE‐ST assessment, capturing data on surgical infrastructure, workforce capacity, patient outcomes, and procedural efficiency [17, 18].

Interpretation and Action Phase: Data were analyzed using descriptive statistics. Each institution received a customized report identifying strengths and opportunities based on predefined thresholds. An automated code was created to generate potential recommendations. Each recommendation assessed key areas across the continuum of care. Thresholds for each recommendation were established based on current evidence‐based practices and expert consensus. An algorithm for each recommendation was developed using the most desirable clinical practices. These algorithms incorporated scores assigned to either a single question or a combination of multiple questions within the tool. When a recommendation was based on multiple questions, a strength was identified only if the predefined threshold was met for all questions included in the algorithm for that specific recommendation. If this condition was not met, the recommendation was categorized as an opportunity.

Each participating institution engaged in a prioritization workshop, where findings were ranked.

After two rounds of ranking of cohort level recommendations, we used an impact–effort matrix to conduct the final prioritization. The matrix allowed participants to collaboratively rate each recommendation on a scale from 1 to 5 according to two criteria: the impact on patient outcomes and the effort required for implementation. Recommendations were then placed within the matrix (see Appendices S1 and S2), enabling participants to develop an action plan that prioritizes projects that are both feasible and likely to yield high impact [19, 20].

### 2.4 Statistical Analysis

Descriptive statistics (frequencies, ranges, and mean scores) were used to summarize performance across modules, domains, and subdomains. Institutions were stratified according to the World Bank income classification (Upper‐Middle‐Income Countries [UMICs] vs. Lower‐Middle‐ and Low‐Income Countries [LMICs/LICs]) for subgroup analysis. Statistical testing was not included due to the limited sample size, which precluded appropriate inferential comparisons.

An aggregated report summarized the frequency of key indicator availability across participating institutions. Opportunities present in more than 50% of institutions were highlighted for discussion during the prioritization workshops to identify cross‐cutting areas for targeted improvement (see Appendices S1 and S2).

## 3 Results

### 3.1 Stages 1 and 2—Construction and Content Validation

PrOFILE‐ST was finalized with 12 modules encompassing 273 questions designed to assess pediatric surgical oncology capacity. Of the original PrOFILE tool, 107 questions were retained, 112 were adapted, and 54 new surgery‐specific questions were added. Content validation by a panel of 10 experts yielded high agreement: the Item‐Content Validity Index (I‐CVI) ranged from 0.78 to 1.0, consistent with accepted standards for small expert panels. Items scoring below the 0.78 threshold were revised or removed. Tool development concluded once no further changes were required and the average scale‐level CVI (S‐CVI/Ave) exceeded 0.90, indicating strong overall content validity.

### 3.2 Stage 3—Pilot Testing

All six institutions completed the Preparation and Assessment phases (Table 1), supported by a user guide, recorded walkthroughs, and optional live support sessions to ensure standardized implementation. One site was unable to complete the Interpretation and Action phase due to political instability.

**TABLE 1:** Pilot Sites Cohort Description (*n* = 6).

Characteristics	*n* (%)
WHO Region	
Africa	33.33
Americas	66.67
World Bank Income Level	
Low income	16.67
Lower middle income	16.67
Upper middle income	66.67
Type of hospital	
Pediatric Hematology/Oncology Hospital	33.33
Cancer Hospital or Institute	16.67
Children's Hospital	33.33
General Hospital	16.67
Other	0
Type of healthcare system	
Public	50
Private, not for profit	50
Private, for profit	0
Combination	0
Government Designation	
Teaching or Training facility	83.33
Referral facility	66.67
Hospital size	
< 15 beds	0
15– 30 beds	0
> 30 beds	100
Newly cancer cases per year^a^	
< 50	16.67
50–100	0
> 100	83.33
Oncologic Surgeries per year^b^	
< 50	0
50– 100	33.33
> 100	66.67
Operating Rooms	
Median	4
Range	(2–11)

Abbreviations: PHO, pediatric hematology and/or oncology; WHO, World Health Organization.

^a^ Only including solid and central nervous system (CNS) tumor patients.

^b^ Excluding biopsies.

**FIGURE 3:**
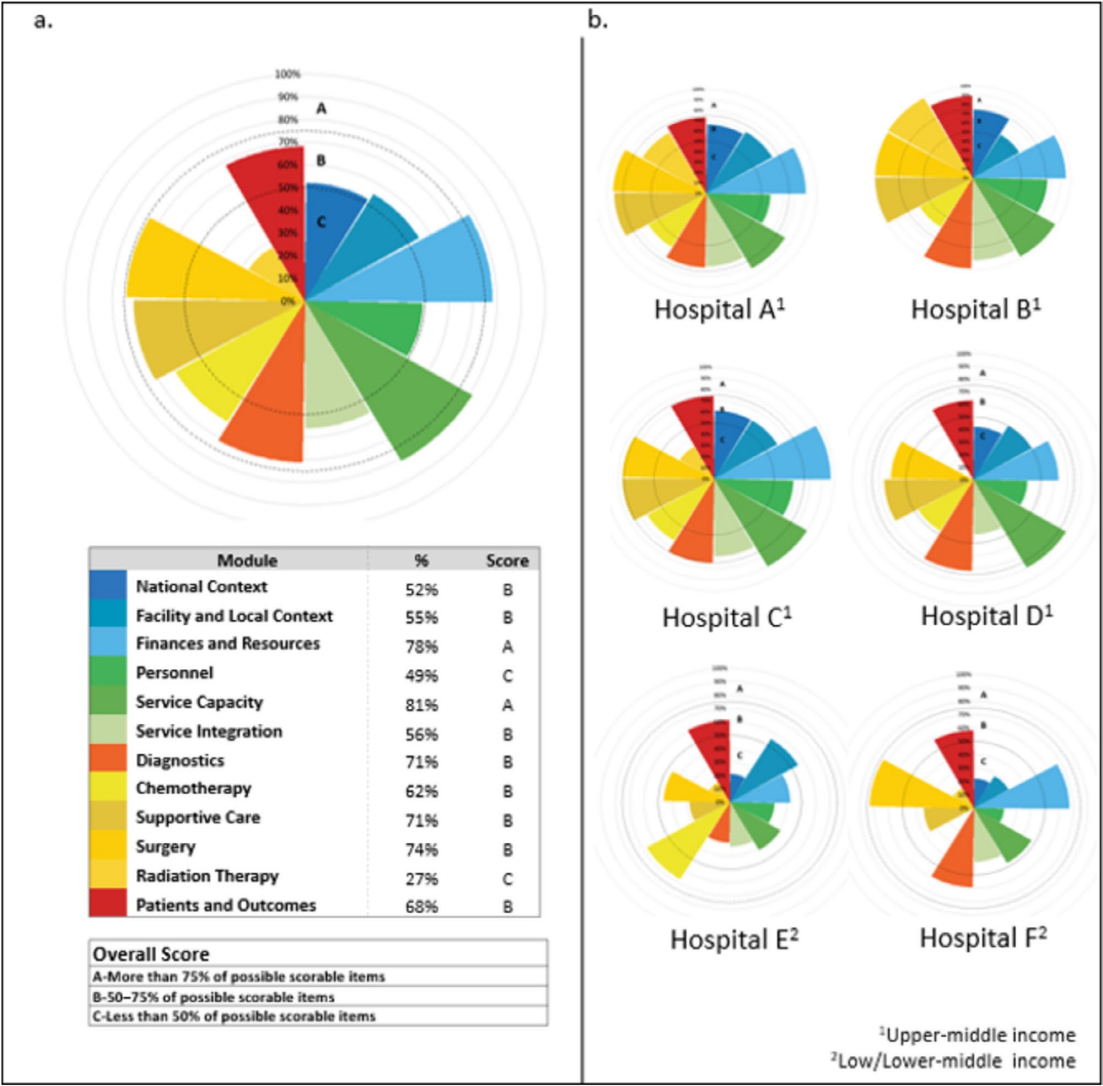
(a) Aggregated and (b) individual polar graphs separated by module.

Institutional polar graphs reflected heterogeneity in performance across modules (Figure 3), but the cohort aggregated polar graph showed that the highest‐scoring modules were Service Capacity (81%) and Finances and Resources (78%), whereas Personnel (49%) and Radiation Therapy (27%) scored lowest. Surgical services ranked third highest overall, underscoring its significance in pediatric oncology care (Figure 3).

### 3.3 Income‐Based Comparisons

Institutions were stratified using the World Bank income classifications to compare performance. Upper‐middle‐income countries (UMICs) demonstrated higher scores in Surgical Services (79.5% vs. 64%) and Surgical Operations (85.5% vs. 44.5%) compared to lower‐middle‐ and low‐income countries (LMICs/LICs). However, LMICs/LICs exhibited strengths in Surgical Operative Reporting (80%) and Surgical Timeliness (65%). While no statistical testing was conducted due to limited sample size, descriptive comparisons revealed shared challenges across income groups, including workforce limitations, fragmented care pathways, and resource constraints (Table 2).

**TABLE 2:** Comparison of Surgery Module and Domain Scoring Results by Income Level.

Domain	Lower middle/Low^a^ (*n* = 2)	Upper middle^b^ (*n* = 4)
Surgery Services	64	79.50
Surgery Operations	44.50	85.50
Surgery Timeliness Associated Factors	65	76.50
Surgery Operative Report	80	93.50
Procedures	64	95
Planning	50	50
Module Total	59	80

^a^ Includes Sudan and Zambia.

^b^ Includes Brazil, Peru, and Mexico.

### 3.4 Prioritization of Quality Improvement Opportunities

Across the cohort, 213 individual opportunities for improvement were identified and consolidated into 13 core priorities through structured ranking. (See Appendices S1 and S2). Using an impact‐effort matrix, expert advisors rated each domain's impact on patient outcomes and effort required for implementation. These inputs informed an impact‐effort matrix used to guide phased quality improvement efforts.

**FIGURE 4:**
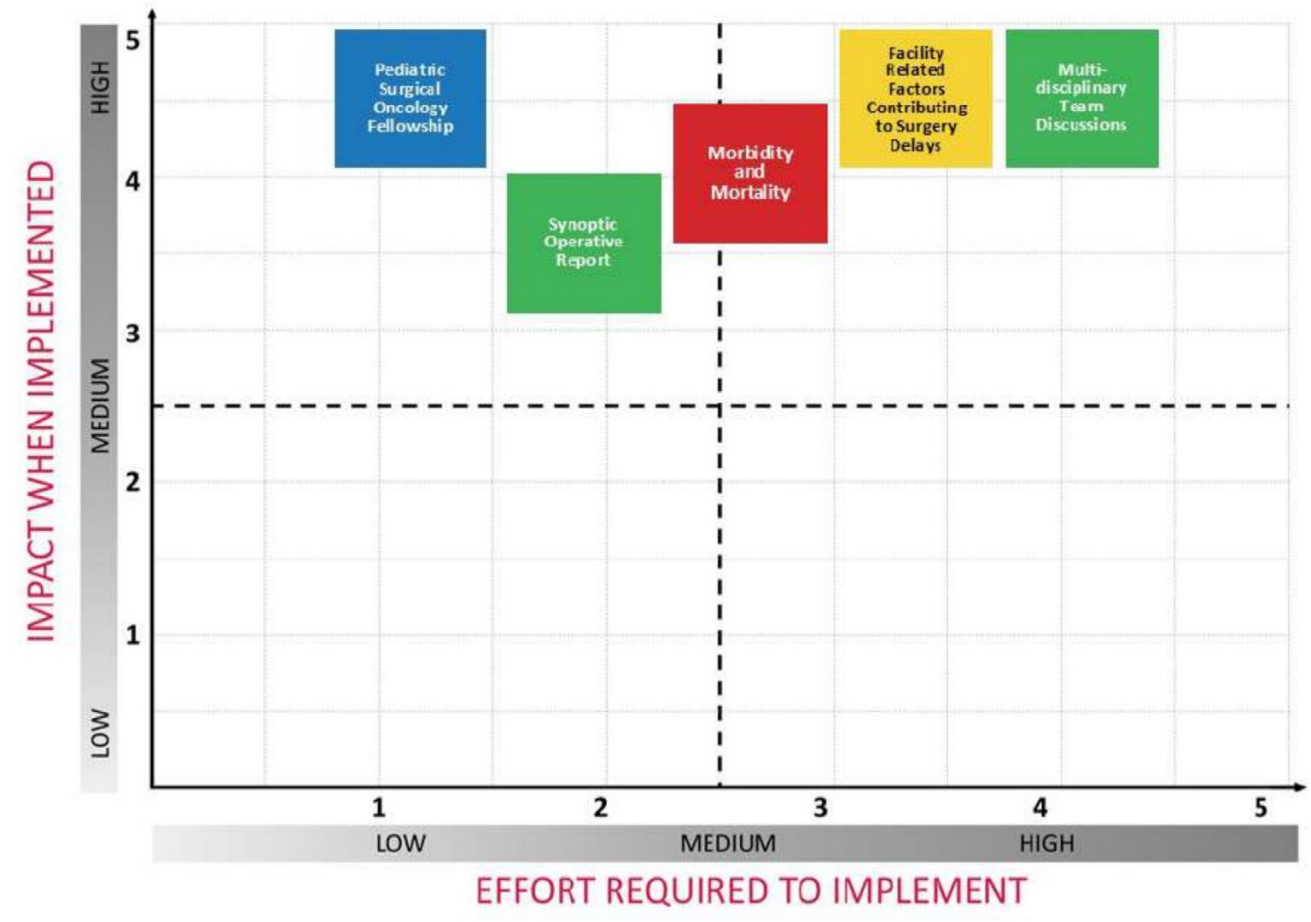
Key areas of opportunity identified at the Prioritization Workshop using an Impact‐Effort Matrix.

Three initiatives were selected for year 1: development of pediatric surgical oncology fellowship, standardization of operative reporting, and the establishment of multidisciplinary team discussions or tumor board. Additionally, two initiatives, including systematic implementation of morbidity and mortality analysis and identification and mitigation of facility‐related factors contributing to surgical delays, were categorized for years 2 and 3 to facilitate phased implementation (Figure 4).

### 3.5 Qualitative Findings

Open‐ended feedback from implementation teams yielded three dominant themes:

### 3.6 Relevance and Value of the Tool

Users described PrOFILE‐ST as timely and comprehensive, filling a critical gap in evaluating surgical oncology capacity in pediatric cancer programs.

### 3.7 Operational Challenges and Enablers

Institutions noted difficulty retrieving data for certain indicators, especially those requiring interdepartmental coordination.

### 3.8 Catalyst for Institutional Dialog

The tool served as a springboard for internal discussions between surgeons, oncologists, and administrators, leading to new quality initiatives and institutional engagement around surgical oncology capacity.

## 4 Discussion

PrOFILE‐ST was successfully implemented across six institutions in five countries, demonstrating its adaptability to diverse healthcare settings. The tool proved to be a feasible and effective means of assessing pediatric oncology surgical care, even in resource‐limited environments. This pilot study confirmed that PrOFILE‐ST provides a reliable framework for evaluating both structural and process‐related aspects of pediatric surgical oncology care, offering a comprehensive assessment across key domains such as service capacity, surgical timeliness, and multidisciplinary team availability.

The pilot testing of PrOFILE‐ST provided critical insights into the capacity and quality of pediatric oncology surgical care in both high‐resource and resource‐limited settings. Performance differences were observed between Upper‐Middle‐Income Countries (UMICs) (Figure 3. Hospitals A, B, C & D) and Lower‐Middle/Low‐Income Countries (LMICs/LICs) (Figure 3. Hospitals E & F) that highlight disparities in surgical infrastructure, service capacity, and timeliness of care that may impact patient outcomes.

UMICs generally demonstrated higher scores in surgical operations and service capacity, likely due to their more advanced infrastructure and greater resource availability. For example, they had better access to surgical tools, advanced imaging, and operating room infrastructure. These observed differences underscore the potential influence of resource allocation on care quality and highlight areas where targeted interventions in LMICs/LICs could help bridge existing gaps.

Despite lower overall scores in LMICs/LICs, certain domains—such as Surgical Operative Reporting and Surgery Timeliness—showed relative strengths. These findings suggest that even in resource‐constrained settings, healthcare teams maintain specific aspects of care, potentially due to the resilience and adaptability of local providers. Strong performance in Surgical Operative Reporting, for instance, indicates that institutions prioritize documentation and standardized reporting despite broader infrastructure and capacity challenges. Furthermore, structured operative reports could serve as a valuable tool for surgeons in diverse settings.

Across all income groups, PrOFILE‐ST identified key opportunities for improvement. Surgical planning and surgical operations were consistently weaker in LMICs/LICs, suggesting that enhancements in these areas could substantially improve overall surgical outcomes. While some institutions demonstrated strong service integration, challenges remained across both income levels. Strengthening multidisciplinary collaboration and integration is essential for optimizing pediatric oncology care.

Several overarching themes emerged as priorities for improvement across all settings, including the need for enhanced surgical training, standardized quality improvement (QI) indicators, and increased multidisciplinary teams' collaboration. Despite institutional heterogeneity, the in‐person workshop yielded common quality improvement project proposals. These findings align with global health priorities in pediatric oncology and provide a roadmap for targeted interventions that potentially improve outcomes in both high‐ and low‐resource settings.

While tools such as the WHO Hospital Assessment Tool provide high‐level assessment of oncology programs infrastructure, they do not specifically evaluate pediatric cancer surgery. Similarly, clinical protocols like the NCCN Harmonized Guidelines and SIOP treatment regimens provide decision‐making guidance but rely on unmeasured assumptions about surgical readiness and performance. PrOFILE‐ST complements these efforts by supplying a validated method to quantify surgical oncology service delivery in children, allowing programs to identify actionable gaps and track improvement over time.

The insights gained from this pilot study offer valuable direction for future global initiatives aimed at enhancing pediatric oncology care. By identifying strengths and areas for improvement, PrOFILE‐ST provides a tailored approach to surgical care enhancement. The tool's ability to generate institution‐specific reports, paired with its structured assessment process, facilitates focused QI efforts that are contextually relevant to each institution's resources and needs [21, 22, 23]. Additionally, PrOFILE‐ST aligns with the WHO's Global Initiative for Childhood Cancer, which aims to improve childhood cancer survival rates, particularly in resource‐limited settings. By assessing nonbiological factors such as service capacity, workforce training, and multidisciplinary collaboration, PrOFILE‐ST directly addresses key components of pediatric oncology care that influence patient outcomes.

PrOFILE‐ST's modular design allows for tailored deployment based on institutional priorities and development, enhancing usability across different health system contexts. Its scoring system and visual summaries enable programs to stratify performance by domain, benchmark against peers, and prioritize improvement efforts based on data. To support prioritization, we incorporated an impact‐effort matrix, which guides institutions to focus on feasible, high‐impact domains for intervention.

In terms of implementation potential, PrOFILE‐ST can be integrated into annual quality improvement (QI) cycles or national pediatric oncology programs. Regular use may support longitudinal tracking of surgical oncology capacity and inform investment in training, infrastructure, and policy. Furthermore, aligning tool deployment with programmatic initiatives like the WHO GICC can help harmonize global pediatric cancer care efforts and strengthen surgical oncology as a critical pillar within multidisciplinary cancer treatment.

Although the pilot study provided valuable insights, several limitations should be acknowledged. The small number of participating institutions may not fully capture the global diversity of pediatric surgical oncology services, limiting the generalizability of the findings. Additionally, the tool's primary focus on institutional factors may overlook other critical elements, such as patient‐level data, that are essential for providing a more comprehensive understanding of surgical outcomes.

Future iterations of PrOFILE‐ST should incorporate refinements based on feedback from this pilot and explore its application in a larger, more diverse group of institutions. Further validation through large‐scale implementation across additional countries and regions will be essential to assess the tool's applicability, reliability, and scalability. Incorporating patient‐level data and clinical outcomes could further enhance PrOFILE‐ST's capacity to guide targeted QI initiatives and improve pediatric oncology surgical care on a global scale.

## 5 Conclusion

Pilot testing of the PrOFILE‐ST has demonstrated that this tool is both feasible and effective in assessing the capacity and quality of pediatric oncology surgery across diverse settings. The results suggest key areas for improvement, particularly in surgical planning, workforce training, and service integration. Moving forward, PrOFILE‐ST has the potential to become a key resource in improving pediatric oncology care, helping institutions identify actionable areas for improvement, monitor progress, and contribute to global efforts to reduce disparities in pediatric cancer outcomes.

## Author Contributions

Simone Abib, Abdelhafeez H. Abdelhafeez, Miriam L. Gonzalez‐Guzman Paola Friedrich, Fernanda de Souza: Conceptualization, methodology, investigation, writing – original draft, supervision. Rodrigo Chaves Ribeiro, Débora Rebollo de Campos, Lily Saldaña Gallo, Raul Ramirez De la Cruz, Jaime Shalkow‐Klincovstein, Jesús Ponce‐Cruz, Mohammed Elnour, Amon Ngongola, Seith Kalota, Bruce Bvulani, Logan Houston, Riya Misal, Heather Forrest, Nadya Sullivan, Alyana Alvarez, Srikanth Naradasu, Aman Maru]: Formal analysis, data curation, writing – review and editing Rodrigo Chaves Ribeiro, Débora Rebollo de Campos, Lily Saldaña Gallo, Raul Ramirez De la Cruz, Jaime Shalkow‐Klincovstein, Jesús Ponce‐Cruz, Mohammed Elnour, Amon Ngongola, Seith Kalota, Bruce Bvulani, Logan Houston, Riya Misal, Heather Forrest, Nadya Sullivan, Alyana Alvarez, Srikanth Naradasu, Aman Maru: Validation, project administration, writing – review and editing Simone Abib, Abdelhafeez H. Abdelhafeez, Miriam L. Gonzalez‐Guzman, Paola Friedrich, Fernanda de Souza, Heather Forrest, Nadya Sullivan, Alyana Alvarez, Srikanth Naradasu, Aman Maru: Visualization, data analysis, writing – review and editing. All authors read and approved the final manuscript.

## Disclosure

Permission to Reproduce Material From Other Sources: No previously published material requiring permission has been included in this manuscript.

## Ethics Statement

This study was conducted in accordance with the ethical principles of the Declaration of Helsinki. Ethical approval was obtained from St. Jude Children's Research Hospital Institutional Review Board and the respective IRBs at all participating institutions.

## Consent

This study did not involve direct patient participation. All data collected were institutional and did not include identifiable patient information, thus waiving the need for individual patient consent.

## Conflicts of Interest

The authors declare no conflicts of interest.

## Supporting information


**Appendix S1.** PrOFILE‐ST PRIORITIZATION WORKSHOP STRUCTURE, AIMS, TOOLS, AND DURATION

**Appendix S2.** PrOFILE‐ST prioritization through iterative ranking and voting.

## Data Availability

The data supporting the findings of this study are available from the corresponding author upon reasonable request.
