# Supplementary material for: Development and Pilot Testing of PrOFILE‐ST: A Pediatric Surgical Oncology Capacity and Quality Assessment Tool for Resource‐Limited Settings
Source: Cancer Med. 2025 Aug 5;14(15):e71122. doi: 10.1002/cam4.71122 (PMC12322827; doi:10.1002/cam4.71122)
Supplement: Supplementary file 1 — Appendix S1. PrOFILE‐ST PRIORITIZATION WORKSHOP STRUCTURE, AIMS, TOOLS, AND DURATION [file CAM4-14-e71122-s002.docx]

**APPENDIX 1 | PrOFILE-ST PRIORITIZATION WORKSHOP STRUCTURE, AIMS, TOOLS AND DURATION**

| **Day** | **Exercise Title** | **Aims** | **Tool** | **Duration** |
| --- | --- | --- | --- | --- |
| 1 | Consensus and Initial Prioritization | Select three opportunities per each PrOFILE-ST module to be carried forward to the prioritization process using a ranking system | Ranking Table | 1 hour 20 minutes |
|  | Final Prioritization | Select three recommendations per each PrOFILE-ST component using the Impact-Effort Matrix | Impact-Effort Matrix | 1 hour 45 minutes |
|  | Categorization | Collaboratively classify opportunities into three categories: Areas that the surgery program is currently working on, Areas that the surgery program is not working on, but important to start and Areas that the surgery program might need to wait and are not feasible to implement at this moment; Create a block diagram that outlines the sequence of steps, timeline, resources needed, and actors involved in the selected recommendations. | Block Diagram | 1 hour 35 minutes |
| 2 | Project Charter | Apply quality improvement methods to one of the selected recommendations from the PrOFILE-ST results; Collaboratively develop an initial project charter for the selected recommendation using IHI Quality Improvement Tools | Aim Statement, Fishbone Diagram, Driver Diagram, Measurement Strategy | 2 hours |
