# Supplementary figures and images for: Development and Pilot Testing of PrOFILE‐ST: A Pediatric Surgical Oncology Capacity and Quality Assessment Tool for Resource‐Limited Settings
Source: Cancer Med. 2025 Aug 5;14(15):e71122. doi: 10.1002/cam4.71122 (PMC12322827; doi:10.1002/cam4.71122)

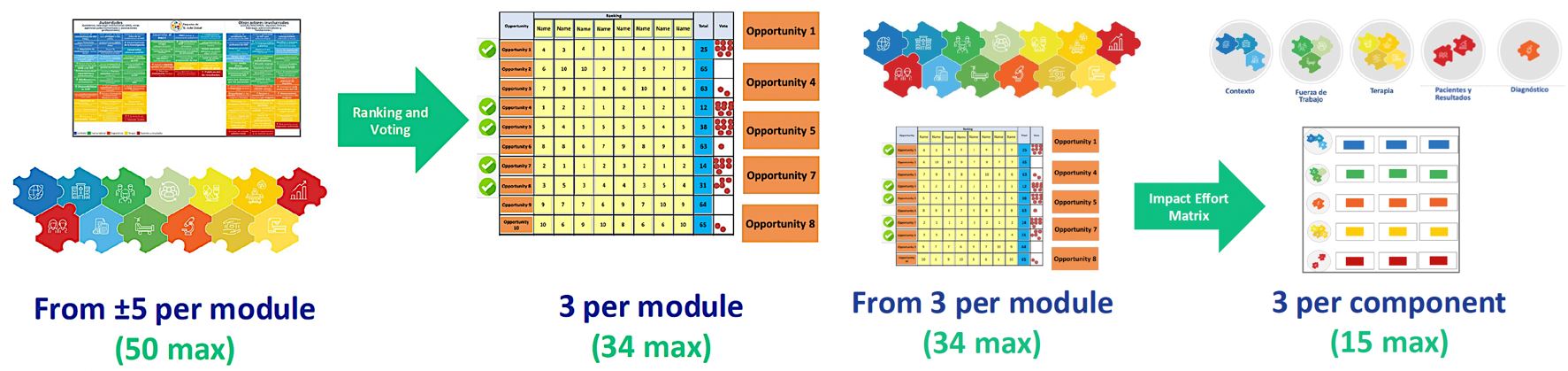

Supplement: Supplementary file 2 — Appendix S2. PrOFILE‐ST prioritization through iterative ranking and voting. [file CAM4-14-e71122-s001.jpg]
